# Hospital Finance

**Published:** 1895-03-16

**Authors:** 


### The Institutional Workshop.

### HOSPITAL FINANCE.

### "SPECIAL HOSPITALS: ROBBERS OF THE POOR"?

On the 23rd ult. we published a communication from a medical correspondent under the above heading. This communication, and especially the title of it, has caused, we think with justice, some misapprehension on the part of the managers of certain worthy special hospitals, and those connected with them, which we much regret. It has been somewhat of a comedy of errors, due in part to the ravages of influenza, which have had no respect for editors, writers, printers, or even the printer's devil. We have suffered, like many other journals, from temporary difficulty of a very acute kind, owing to the inconsiderate completeness of the work done amongst us by the prevailing epidemic. It was clearly indicated, for instance, on the proof passed for the press that the communication in question was "from a correspondent." But, alas! the influenza upset the composing-room that evening, and so the current week's edition came out like a zebra marked with many stripes. Then again, our correspondent took the influenza, and in revising his proof with the "flue" thick upon him omitted to make it perfectly clear, as he intended to do, that there are special hospitals and special hospitals, and that the best special hospitals rival, if they do not even surpass, some general hospitals in the excellence of their administration and the value of the work they do for the poor. Our correspondent did not, we are confident, intend to hit any but the bad, unnecessary, ill-managed hospitals, of which there are, alas! still some existent in London naked and unashamed.

The editor being now convalescent, and the enemy in full retreat, he begs to assure his correspondents and readers that the policy of The Hospital is unchanged and unaltered towards special hospitals, and that it stands where it did on the solid rock of efficiency and utility. Many special hospitals are still invaluable to the public and the profession. Amongst them those for the Paralysed and Epileptic, the Lock, Lying-in, Women, Children, Fever, Consumption, and others, many of which are as economically, if not better, managed than any other institutions in the country, as readers of "Burdett's Hospital and Charities Annual" can see for themselves. In these circumstances, while appreciating the zeal and interest which are the distinguishing attributes of all connected with special hospitals, we hope our assurance of continued appreciation and support of every worthy special hospital will remove every misapprehension, and dispel the illusions which the influenza has successfully caused in this, as in many other cases, where it has obtruded its hateful attentions.

